# The Role of the Neighborhood Social Environment in Physical Activity among Hispanic Children: Moderation by Cultural Factors and Mediation by Neighborhood Norms

**DOI:** 10.3390/ijerph17249527

**Published:** 2020-12-19

**Authors:** Yeonwoo Kim, Lorrene Ritchie, Andrew Landgraf, Rebecca E. Hasson, Natalie Colabianchi

**Affiliations:** 1 Department of Kinesiology, The University of Texas at Arlington, Arlington, TX 76010, USA; yeonwoo.kim@uta.edu; 2 Nutrition Policy Institute, Division of Agriculture and Natural Resources, University of California, Oakland, CA 94607, USA; lritchie@ucanr.edu; 3 Battelle Memorial Institute, Columbus, OH 43201, USA; andland@gmail.com; 4 School of Kinesiology, University of Michigan, Ann Arbor, MI 48109, USA; hassonr@umich.edu; 5 Institute for Social Research, University of Michigan, Ann Arbor, MI 48104, USA

**Keywords:** social environment, cultural factors, physical activity, neighborhood social norms

## Abstract

Little is known about how the neighborhood social environment (e.g., safety, crime, traffic) impacts child physical activity. We examine the mechanism by which the neighborhood social environment is associated with child physical activity, moderated by individual-level cultural factors (e.g., language at home, immigrant generation) and mediated by neighborhood physical activity-related social norms (e.g., seeing walkers in the neighborhood). Data included 2749 non-Hispanic White and Hispanic children from the Healthy Communities Study. Multilevel regression was performed. The neighborhood social environment was not associated with physical activity in the full sample. However, Hispanic children speaking both English and Spanish and first- or second-generation Hispanic children engaged in more physical activity when the quality of the neighborhood social environment was higher (*b* = 1.60, *p* < 0.001 for Hispanic children speaking English and Spanish; *b* = 2.03, *p* < 0.01 for first-generation Hispanic children; *b* = 1.29, *p* < 0.01 for second-generation Hispanic children). Neighborhood physical activity-related social norms mediated the association between the neighborhood social environment and physical activity among Hispanic children speaking English and Spanish (*b* = 0.33, *p* < 0.001) and second-generation Hispanic children (*b* = 0.40, *p* < 0.001). Findings suggest heterogeneity in how neighborhood social environments impact physical activity by cultural factors. Health promotion programs may need to enhance neighborhood social environments to increase Hispanic children’s physical activity.

## 1. Introduction

Lack of physical activity among children is a serious public health issue in the United States (US). Only four in ten US children engage in the recommended amount of physical activity [1], and 11% of children do not participate in any vigorous physical activity in a given week [2]. When examining physical inactivity by ethnicity, Hispanic children are the most inactive; 15% of Hispanic children with US-born parents, and 17–23% of Hispanic children with immigrant parents do not engage in any vigorous physical activity (vs. 9% of non-Hispanic White children) [2]. A low level of physical activity among Hispanic children contributes to their high prevalence of overweight and obesity (over 23%) [3], a prevalence that has continued to increase despite recent declines among non-Hispanic White children [4]. As Hispanic populations are the largest ethnic minority group in the US, representing 29% of children [5], and will account for 32% of the total child population in 2060 [6], attention has recently increased to identify factors related to physical inactivity in Hispanic children. 

### 1.1. Neighborhood Impacts on Child Physical Activity

The neighborhood environment can affect child physical activity levels, for example, by influencing whether they can safely walk to a park or playground [7,8,9,10]. In addition, children may observe more residents engaging in physical activity when living in safe and secure neighborhoods. The active lifestyle of residents attributes to social norms, which may encourage children to engage in physical activity [11]. In other words, living in safe and secure neighborhoods may positively affect child physical activity, mediated by social norms around engaging in activity. Empirical studies that have investigated the association between the neighborhood social environment (e.g., safety, crime, traffic) and child physical activity have reported mixed results [8,9,12,13,14,15,16,17]. The mixed findings may partly be explained by limited consideration of moderating factors such as individual-level ethnicity and cultural factors (e.g., immigrant generation, language spoken at home), in addition to variation in survey designs, sample characteristics, and measurements. Thus, the present study examines the moderating effect of individual-level cultural factors in the association between the neighborhood social environment and physical activity. In addition, given that no prior studies have investigated potential mediating mechanisms underlying an association, this study investigates the mediating role of neighborhood physical activity-related social norms in the association between the social environment and child physical activity. 

### 1.2. Considering Cultural Factors to Understand How Neighborhood Impacts Physical Activity

Several studies have examined individual-level cultural factors, such as language spoken at home and immigrant generation, as a correlate of physical activity in Hispanic children [2,17,18,19,20,21,22,23]. Theoretically, two contrasting mechanisms have been proposed to examine the association between cultural factors and physical activity in Hispanic children. On the one hand, as physical activity is often a social behavior for children mostly involving groups of peers [24,25], fewer language barriers and greater exposure to US culture are likely to provide more opportunities to be physically active with peers from diverse cultural backgrounds. On the other hand, greater exposure to US culture may increase the risk of inactivity for Hispanic children given acculturation-related stress (leading to unhealthy lifestyle) and the high levels of physical inactivity in the US [26,27,28]. A few empirical studies reported that being first-generation or Spanish speaking is directly associated with less physical activity among Hispanic children after adjusting for parental education and economic level [2,20,21], which supports the first conceptual mechanism (see an exception in [18]). 

Cultural factors may interact with social environments to affect child physical activity although to our knowledge, no empirical research has investigated its interaction effect. Past literature showed variation across Hispanic immigrant generations in types of resources that parents use to support their children. For example, parents of first-generation Hispanic children used informal support for their children, while parents of third-generation Hispanic children used formal and structural support for their children [29]. Living in a high-quality social environment may be an important informal support for Spanish-speaking Hispanic children and first- or second-generation Hispanic children. Safe and supportive environments may lead to more physical activity within the neighborhood thereby exposing them to visual cues of neighbors and peers being physically active in their neighborhood. Also, Spanish-speaking or first- or second-generation Hispanic families can face practical and cultural challenges to navigating and utilizing physical activity resources outside their neighborhood [21,30,31,32]. Thus, living in a health-promoting neighborhood may provide more beneficial informal support for them in comparison to non-Hispanic White children and Hispanic children from English-speaking or third-generation families who may be more likely to use formal and structural resources. 

### 1.3. The Present Study

The present study extends past research by examining moderating and mediating mechanisms underlying the association between the social environment and physical activity in non-Hispanic White and Hispanic children. We hypothesized that (1) the protective role of the social environment in physical activity would be stronger for Spanish-speaking or first- or second-generation Hispanic children compared to non-Hispanic White children and English-speaking or third-generation Hispanic children; and (2) active and healthy social norms in the neighborhood would mediate the association between the social environment and physical activity. 

## 2. Methods 

### 2.1. Participants

We used data from the Healthy Communities Study (HCS; 2013–2015). The Battelle Memorial Institute and its academic partners (University of California at Berkeley, University of South Carolina, and University of Kansas) designed and conducted the HCS, an observational study designed to examine community impacts on child obesity in the US. The HCS first recruited a probability-based sample of 102 communities (defined by high school catchment areas) by stratifying by race, ethnicity, income, region, and a pre-selection score of program and policy intensity [33]. The HCS added 28 communities that were purposefully selected for their childhood obesity prevention efforts [33]. The HCS recruited child-parent/guardian dyads (n = 5138) from elementary and middle schools located in 130 communities. Trained personnel collected the data from participants at home visits including socio-demographic information, physical activity behavior, and child perception of their social environment. The respondent was determined by child age. Specifically, child age was recorded, and the Computer-Assisted Interview system guided who was to answer each question. Additional details about the HCS have been reported elsewhere [33]. 

Our research team obtained access to the HCS data under contract and extended the HCS data by linking the American Community Survey 2009–2013 data to the HCS participant data. Our sample included non-Hispanic White children (US-born and English-speaking at home) and Hispanic children who lived at their current address for 1 year or more and had a geocoded address (n = 3472). We excluded Hispanic children who used language other than English and/or Spanish due to the small sample size (n = 9), resulting in 3463 children. Participants were excluded if they had missing data on: physical activity (n = 193), child’s or parents’ country of birth (n = 269), two or more items for child perception of the social environment (n = 39), neighborhood physical activity-related social norms (n = 47), maximum parent education (n = 14), and household income (n = 142). The resulting final sample included 2749 child-adult respondent dyads. Compared with respondents remaining in the study, those excluded were more likely to be older, Hispanic ethnicity, in families with lower socioeconomic status, and have lower levels of child-perceived social environment. There were no significant differences (*p* > 0.05) between included and excluded samples in physical activity level and neighborhood physical activity-related norms. This study was reviewed by the University of Michigan Institutional Review Board and the Battelle Memorial Institute Institutional Review Board (The University of Michigan’s IRB HUM number is HUM00138571. The Battelle Memorial Institute’s IRB number is IRB 0677-100112203 Rev 0.0). 

### 2.2. Measures

#### 2.2.1. Outcome Measure

Physical activity was measured using the 7-day Physical Activity Behavior Recall (PABR-7) instrument designed to elicit participation in 14 types of activities in the prior week. We used six of the 14 activity types including pick-up sports (e.g., basketball, football), non-school sports, physically active games, swimming, outdoor/adventure activities (e.g., rock climbing), and walk/bike for fun/exercise. We excluded the other eight activity types that were performed at school because we focused on associations with residential neighborhood-based physical activity. Using a computer-assisted interview, respondents indicated the days on which the child did each activity during the past week. These questions were answered by an adult respondent (mostly parent) for children aged 4–8; otherwise by the child participant for children aged 9–15. Physical activity was examined as a continuous variable (sum of number of times in the past week for all 6 physical activities). Additional details on physical activity measures are available elsewhere [34].

#### 2.2.2. Independent Variable

Child perception of the social environment was included as an independent variable. Children responded to a four-item scale each with four response options (disagree a lot to agree a lot): “It is safe to walk or jog in the neighborhood during the day,” “There is so much traffic that it makes it hard to walk in the neighborhood (reverse coded),” “There is a lot of crime in the neighborhood (reverse coded),” and “There are lots of loose or scary dogs in the neighborhood (reverse coded).” We calculated the average of four items (range 1–4 points) allowing for one item to be missing. For children aged 4–11, an adult respondent (mostly parent) assisted the child to answer the questions. Higher scores represent a better social environment (Cronbach’s alpha = 0.60). 

#### 2.2.3. Moderators

Moderators included individual-level cultural factors, which were measured using ethnicity, language at home and immigrant generation. First, language at home was measured based on Hispanic identification and language spoken at home. Adult respondents were asked the focal child’s Hispanic identification (Hispanic or not) and language spoken at home. English-speaking non-Hispanic White children were coded as “English-speaking non-Hispanic White children” (=0). If the Hispanic child spoke only English at home, the child was coded as “Hispanic children speaking English” (=1). If the Hispanic child used English and Spanish equally or more English than Spanish at home, the child was coded as “Hispanic children speaking English and Spanish” (=2). If the Hispanic child spoke Spanish only or more than English, the child was coded as “Hispanic children speaking Spanish” (=3). Second, immigrant generation was measured based on the country of birth for the children and their biological parents. The variable was categorized as “US-born non-Hispanic White children” (=0), “first-generation Hispanic children” (=1), “second-generation Hispanic children” (=2), and “third- or higher-generation Hispanic children” (=3).

#### 2.2.4. Mediator

Neighborhood physical activity-related social norms were included as a mediator between the social environment and physical activity. Neighborhood physical activity-related social norms were measured as a continuous variable based on two questions asked of children with four response options (disagree a lot to agree a lot): (1) people in my neighborhood can easily see walkers and bikers on the streets from their homes; and (2) I often see other girls or boys playing outdoors in my neighborhood. 

#### 2.2.5. Covariates

Covariates included child age, child sex, biological parents’ highest education, annual household income, and neighborhood socioeconomic status. We used the American Community Survey 2009–2013 to obtain the measure of neighborhood socioeconomic status. Neighborhood socioeconomic status was measured by summing the z scores of variables representing income, housing values, education, and occupation at the block group level and then calculating the weighted average of the summated z scores within 1 km of the participant’s residence [35,36]. We categorized neighborhoods into three groups using tertiles of weighted neighborhood socioeconomic status score. 

### 2.3. Analysis

Descriptive statistics and analysis of variance (ANOVA) were used to explore the sample characteristics and the bivariate relationship between sociodemographic characteristics and child physical activity level. In subsequent regression analysis, multilevel modeling was used given that the data from the HCS were clustered at the community level (intraclass correlation for physical activity = 6.46%). We first conducted a multilevel linear regression model to assess associations between the social environment and physical activity in non-Hispanic White and Hispanic children. Second, we tested two interaction terms, one at a time, on the association between the social environment and physical activity: (1) interaction term of social environment and language at home and (2) interaction term of the social environment and immigrant generation. Due to the significant differences of some variables between the excluded sample and the included sample, we conducted multiple imputation for missing data among 3463 children and assessed multilevel linear regression models as a sensitivity analysis. Lastly, we conducted a structural equation model using bootstrap analyses (500 bootstrap samples) to test the mediation effect of neighborhood physical activity-related social norms in the association between the social environment and physical activity [37]. We decomposed the total, direct, and indirect effects to further understand the direction and extent of the mediation effect. When a cultural factor was a significant moderator in the association between the social environment and physical activity, the mediation analysis was conducted in the groups where the social environment was significantly associated with physical activity. Analyses were performed using Stata Version 15 (StataCorp LLC, College Station, TX, USA). 

## 3. Results

Sample characteristics are shown in Table 1. In the analytical sample, 44% of children identified as English-speaking non-Hispanic White children, 36% identified as Hispanic children speaking English and Spanish, 14% identified as Hispanic children speaking Spanish, and 6% identified as Hispanic children speaking English at home. Regarding immigrant generation, 44% of children were second-generation Hispanic children, 7% identified as first-generation Hispanic children, and 6% identified third- or higher-generation Hispanic children. Children reported participating in any of six physical activity types 7.8 times during the past week on average. 

Table 2 present the results of bivariate analyses. Bivariate statistics showed a significant difference in physical activity level between the following groups: (1) children aged 10–12 years (M = 8.2) and children aged 13–15 years (Mean = 7.1), (2) boys (Mean = 8.1) and girls (Mean = 7.4), (3) English-speaking non-Hispanic White children (Mean = 8.4) and Hispanic children speaking English and Spanish or speaking Spanish (Mean = 7.2 and 7.1, respectively), (4) US-born non-Hispanic White children (Mean = 8.4) and second generation Hispanic children (Mean = 7.2), (5) children of parents with some college (Mean = 8.0) and children of parents with less than high school (Mean = 7.0), (6) children of parents with college graduate or above (Mean = 8.3) and children of parents with less than high school (Mean = 7.0), (7) children of families with annual income $75,001 or above (Mean = 8.4) and children of families with annual income less than $20,000 (Mean = 7.3), $20,000–35,000 (Mean = 7.3), and $35,001–50,000 (Mean = 7.3), and (8) children living in high SES neighborhoods (Mean = 8.3) and children living in low or moderate SES neighborhoods (Mean = 7.6 and 7.3, respectively).

**Figure 1:**
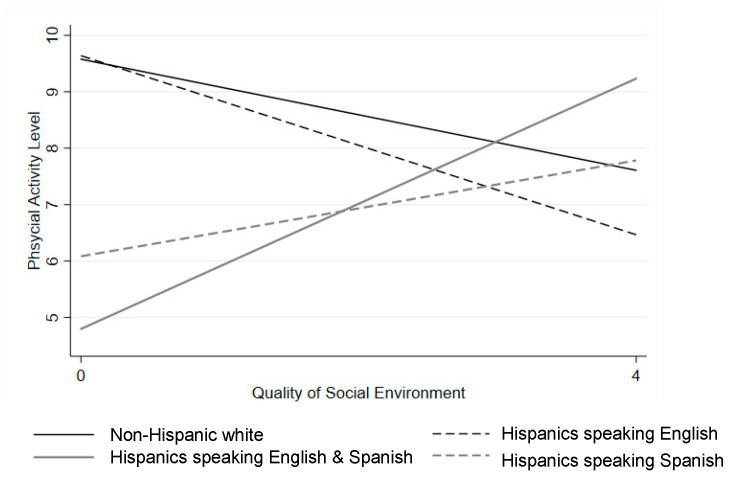
Predicted physical activity associated with the social environment by child language at home, as shown in Table 3, Model 3, setting all other variables within the model at their average value. Note. The social environment was measured based on a four-item scale on neighborhood safety, traffic, and crime. Higher scores indicate higher quality social environment.

Table 3 presents associations of the social environment and language at home with physical activity. The social environment was not significantly associated with physical activity in the overall sample. Hispanic children speaking English and Spanish or speaking Spanish had less physical activity than non-Hispanic White children (b = −1.38 and −1.66, respectively, *p* < 0.001) after controlling for child age and gender (Model 1). With the inclusion of parental education, household income, social environment, and neighborhood socioeconomic status in the model (Model 2), the associations remained significant (b = −0.94, *p* < 0.05 and b = −1.24, *p* < 0.01 respectively). There was no statistical difference in physical activity between Hispanic children speaking English and non-Hispanic White children (b = −0.62, *p* > 0.05). In Model 3, there was a significant interaction effect of speaking English and Spanish at home (vs. non-Hispanic White children) in the association between the social environment and physical activity (b = 1.60, *p* < 0.001). As shown in Figure 1, the association between the social environment and physical activity was positive for Hispanic children speaking English and Spanish whereas the association was negative and flatter for non-Hispanic White children. Although it was not significant at alpha = 0.05 for Hispanic children speaking Spanish, the association between the social environment and physical activity was marginally significant and positive for Hispanic children speaking Spanish in comparison to non-Hispanic White children (b = 0.92, *p* < 0.10). 

**Figure 2:**
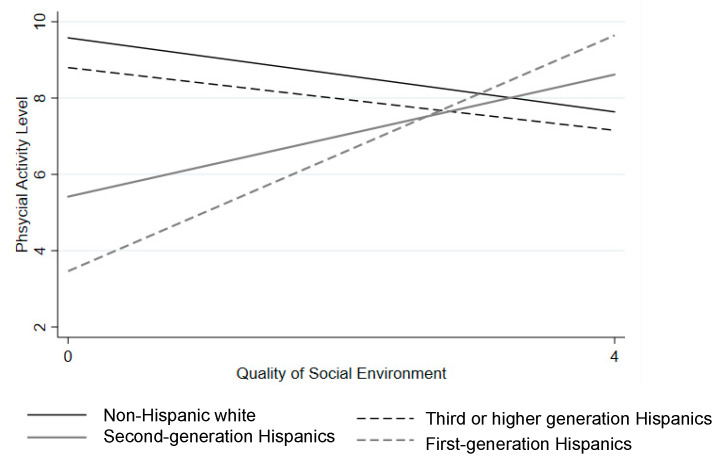
Predicted physical activity associated with the social environment by child immigrant generation, as shown in Table 4, Model 3, setting all other variables within the model at their average value. Note. The social environment was measured based on a four-item scale on neighborhood safety, traffic, and crime. Higher scores indicate higher quality social environment.

Table 4 presents associations of the social environment and immigrant generation with physical activity. The social environment was not significantly associated with physical activity in the overall sample. First or second-generation Hispanics had less physical activity than US-born non-Hispanic White children after adjusting for child age and gender (b = −1.53, *p* < 0.01 and b = −1.46, *p* < 0.001 respectively). The associations remained significant (b = −1.12, *p* < 0.05 for first-generation Hispanics and b = −1.03, *p* < 0.01 for second-generation Hispanics) when parental education, household income, social environment, and neighborhood socioeconomic status were added to the model (Model 2). There was no significant difference in physical activity between third- or higher-generation Hispanics and non-Hispanic White children. In Model 3, significant interactions were found between the social environment and child immigrant generation in the association with physical activity. As shown in Figure 2, the association between the social environment and physical activity was positive for first- and second-generation Hispanic children (b = 2.03, *p* < 0.01 and b = 1.29, *p* < 0.01, respectively), whereas the association was negative and flatter for non-Hispanic White children.

As a sensitivity analysis, we conducted multiple imputation for missing data and examined the interaction effect of cultural factors in the association between the social environment and physical activity in the imputed data. Table 5 presents pooled estimates after multiple imputation. Multiple imputation changed the magnitude of coefficients but did not alter the significance. 

**Figure 3:**
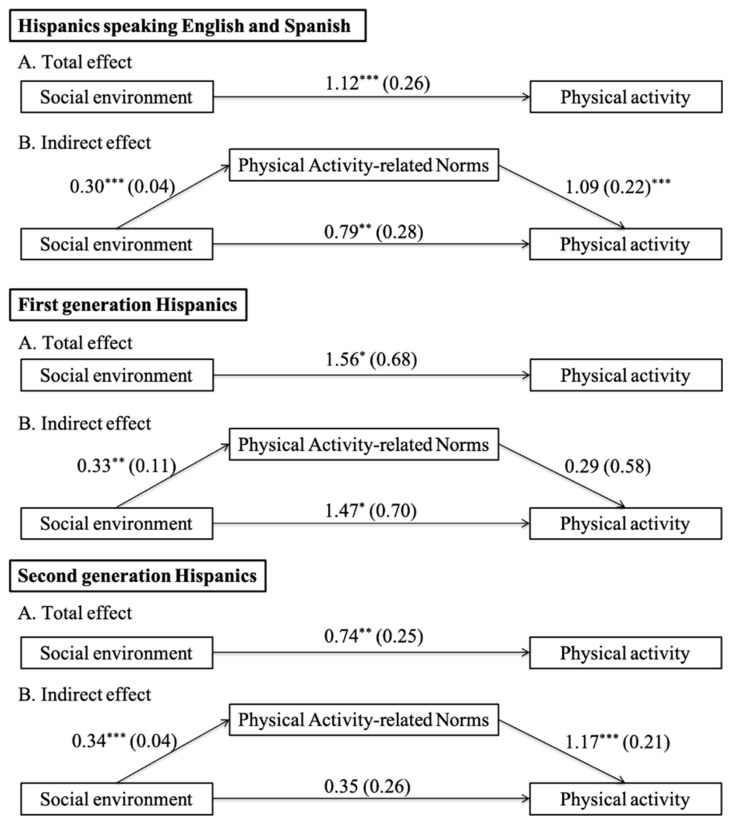
Path diagram for the effect of the social environment on physical activity after bootstrapping. * *p* < 0.05, ** *p* < 0.01, *** *p* < 0.001. Note: Numbers indicate unstandardized regression coefficients and standard errors in parentheses. A represents the total effect of the social environment on frequency of physical activity, and B represents the indirect effect of the social environment on frequency of physical activity through physical activity-related norms. The social environment was measured based on a four-item scale on neighborhood safety, traffic, and crime. Neighborhood physical activity-related social norms were measured based on child-reported indicators whether seeing people being active on the streets. Higher scores indicate higher quality social environment or more physical activity-promoting norms. All analyses were conducted with adjustment for covariates listed in Table 3 and Table 4.

Finally, we tested the mediation of neighborhood physical activity-related social norms in the association between the social environment and physical activity. Before the mediation analysis was performed, we stratified the data by two cultural factors and tested the association between the social environment and physical activity in the stratified models. As previously reported, the association was significant in Hispanic children speaking English and Spanish, first-generation Hispanic children, and second-generation Hispanic children, but not in non-Hispanic White children, Hispanic children speaking English or Spanish, and third- or higher-generation Hispanic children. Thus, mediation analysis was performed in Hispanic children speaking English and Spanish, first-generation Hispanic children, and second-generation Hispanic children, separately. Table 6 and Figure 3 present the estimates of the effects of the social environment on physical activity. As shown in Table 6, the indirect effect estimates indicate a significant mediating role of neighborhood physical activity-related social norms for Hispanic children speaking English and Spanish (b = 0.33 [=0.30 × 1.09], *p* < 0.001) and second-generation Hispanic children (b = 0.40 [=0.34 × 1.17], *p* < 0.001). There was no significant mediation effect of neighborhood physical activity-related social norms for first-generation Hispanic children (b = 0.09[=0.33 × 0.29], *p >* 0.05).

## 4. Discussion

The present study aims to extend current knowledge by investigating mediating and moderating mechanisms by which the neighborhood social environment is associated with physical activity among non-Hispanic White children and Hispanic children. In the full sample of non-Hispanic White children and Hispanic children, the social environment was not significantly associated with physical activity. However, the association in the full sample masked subgroup differences by individual-level cultural factors. We observed that Hispanic children speaking both Spanish and English, first-generation Hispanic children, and second-generation Hispanic children had greater physical activity when living in neighborhoods with high quality social environments (vs. neighborhoods with the lowest quality social environments). For example, Hispanic children speaking Spanish and English engaged in greater physical activity by 1.11 times a week (1.11 = −0.49 + 1.60) for each unit increase in the social environment. When living in neighborhoods with the highest quality social environment, Hispanic children speaking Spanish and English showed greater physical activity than non-Hispanic White children by 1.62 times a week (1.62 = −4.78 + [1.60 × 4 units]). Similar results were found in the interaction effect of immigrant generation in the association between the social environment and physical activity—first- and second-generation Hispanic children reported greater physical activity by 1.55 times a week (1.55 = −0.48 + 2.03) and 0.81 times a week (0.81 = −0.48 + 1.29) for each unit increase in the social environment, respectively. The results are aligned with prior research suggesting that informal support may be especially important for Hispanic children given that their families prefer to use informal support over formal support [29]. Families of first- and second-generation Hispanic children and Hispanic children speaking Spanish and English at home may experience challenges with navigating and utilizing formal support outside their neighborhood [21,30,31,32]. Thus, living in health-promoting neighborhoods may provide more beneficial informal support for them in comparison to non-Hispanic White children and Hispanic children from English-speaking or third-generation families who may be more likely to use formal resources. Based on these findings, we suggest that community policies focusing on change in the quality of the social environment may have a spillover influence on increasing physical activity behaviors in first- and second-generation Hispanic children and Hispanic children speaking Spanish and English. 

Our results of mediation analysis showed that neighborhood physical activity-related social norms mediate the association between the social environment and physical activity in Hispanic children speaking Spanish and English and second-generation Hispanic children (Table 6 and Figure 3). It is possible that visual cues of observing neighbors and peers physically active in their neighborhoods reduce fears of exercising and leads children to accept such active, healthy lifestyles as the norm. The findings suggest that health promotion policymakers need to acknowledge the protective effect of the social environment in physical activity among Hispanic children speaking Spanish and English and second-generation Hispanic children. Also, structured neighborhood physical activity programs may help to increase physical activity participation in Hispanic children speaking Spanish and English and second-generation Hispanic children. 

On the other hand, we did not observe a statistically significant mediation effect for first-generation Hispanic children. The underpinnings of our observed non-significant mediation effect for the group are not clear; one possibility is due to a small sample size issue (n = 180 for first-generation Hispanic children). Also, neighborhood experiences may be different across the strength of ties to Hispanic culture. First-generation Hispanic children who still hold stronger ties to Hispanic culture may have different neighborhood experiences according to the proportion of Hispanic population in the neighborhood, which was not adequately captured in this study. Future research should examine additional features of the neighborhood environment (e.g., racial/ethnic composition, social cohesion, social support, built environment) and if specific features mediate the effect of the social environment on physical activity for first-generation Hispanic children. 

Important strengths of the present study include geographical diversity in the sample. In addition, Hispanic communities and families were oversampled, which led to a large number of Hispanic children in the study (1538 out of 2749 children). The main strength of our study is the investigation of the mediating and moderating mechanisms by which the neighborhood social environment is associated with physical activity, which have not been empirically tested. The potential public health significance is great given the large ethnic disparities in child physical activity in the US and the serious potential consequences of physical inactivity on child health. Our study is also subject to notable limitations. We used a cross-sectional design which prevents us from inferring causality. Due to the cross-sectional design, there is a possibility of reverse causation in which those physically active tend to choose to live in neighborhoods with high quality social environments. Longitudinal designs would elucidate the causal relation and capture the length of exposure to the neighborhood social environment and changes in the neighborhood social environment caused by residential mobility. Also, study findings are not generalizable to all US children because the HCS is not a nationally representative study despite being national in scope. Physical activity was measured using the PABR-7 instrument. The PABR-7 instrument was designed for the HCS and has been used in associational studies [36,38], but the psychometric properties have not been established [34]. Although we excluded any item explicitly asking about physical activity at school, whether physical activity occurred in a child’s residential neighborhood was not measured. In addition, our study did not include individual-level psychological factors such as self-efficacy, motivation, and social desirability [39]. Finally, there are other factors that were not included in this analysis that may affect physical activity, such as parents’ physical activity, spending time with family, psychological stress, language barriers, acculturation, enculturation, and discriminative experiences [40,41]. Further research should explore these various characteristics as determinants of physical activity.

## 5. Conclusions

This study showed that physical activity level varies within Hispanic children by cultural factors, and that a high-quality neighborhood social environment may serve as a resource to increase physical activity among Hispanic children speaking both Spanish and English and first- or second-generation Hispanic children. We suggest that health promotion policymakers should focus on improving the quality of neighborhood social environments as a means to encourage physical activity in children from first- and second-generation Hispanic families and those from Hispanic families speaking Spanish and English. Also, our results demonstrated that neighborhood physical activity-related social norms mediate the association between the neighborhood social environment and child physical activity in second-generation Hispanic children and Hispanic children speaking English and Spanish. The results imply that second-generation Hispanic children and Hispanic children speaking English and Spanish benefit from safe neighborhoods and from healthy social norms around physical activity. Community health programs might need to promote healthy neighborhood norms to increase physical activity in these children. Future research is warranted to include a variety of characteristics such as acculturation, discrimination, neighborhood racial/ethnic composition, and psychological factors to further understand the mechanism by which the social environment impacts physical activity in Hispanic children.

## Figures and Tables

**Table 1:** Sample characteristics, n = 2749.

Characteristic	n	%
Frequency of physical activity (Mean times per week ± SD)	7.8 ± 6.1	
Child age		
4–6 years	528	19.2
7–9 years	978	35.6
10–12 years	863	31.4
13–15 years	380	13.8
Child gender		
Boy	1400	50.9
Girl	1349	49.1
Child language at home		
Non-Hispanic White ^a^	1211	44.1
Hispanics speaking English	160	5.8
Hispanics speaking English and Spanish	987	35.9
Hispanics speaking Spanish	391	14.2
Child immigrant generation		
Non-Hispanic White ^a^	1211	44.1
First generation Hispanics	180	6.6
Second generation Hispanics	1200	43.7
Third or higher generation Hispanics	158	5.8
Maximum parent education		
Less than high school	742	27.0
High-school graduate	455	16.6
Some college	572	20.8
College graduate or above	980	35.7
Annual household income		
Less than $20,000	634	23.1
$20,000–35,000	633	23.0
$35,001–50,000	346	12.6
$50,001–75,000	303	11.0
$75,001 or above	833	30.3
Child-perceived social environment (Mean ± SD) ^b^	2.5 ± 0.7	
Neighborhood physical activity social norms (Mean ± SD) ^b^	3.1 ± 0.8	
Neighborhood socioeconomic status ^c^		
Low SES (<−0.40)	902	32.8
Moderate SES (−0.40 to −0.02)	841	30.6
High SES (>−0.02)	1006	36.6

^a^ English-speaking, US-born non-Hispanic White children. ^b^ Social environment was measured based on a four-item scale on neighborhood safety, traffic, and crime. Neighborhood physical activity-related social norms were measured based on two indicators of seeing others being active in the neighborhood. Higher scores indicate higher quality social environment or more physical activity-promoting norms. ^c^ Neighborhood socioeconomic status was based on American Community Survey 5 year estimates of 2009–2013. We categorized this variable into three groups by tertiles. Higher tertiles represent higher socioeconomic status.

**Table 2:** Bivariate statistics, n = 2749.

Characteristics	Average Frequency of Physical Activity	
	Mean ± SD ^c^	F (t-Value)
Child age		3.64 *
4–6 years	7.5 ± 5.4	
7–9 years	7.8 ± 5.9	
10–12 years	8.2 ± 6.5	
13–15 years	7.1 ± 6.0	
Child gender		(t = 2.86 **)
Boy	8.1 ± 0.2	
Girl	7.4 ± 0.2	
Child language at home		8.71 ***
Non-Hispanic White ^a^	8.4 ± 6.0	
Hispanics speaking English	7.8 ±5.8	
Hispanics speaking English and Spanish	7.2 ± 6.1	
Hispanics speaking Spanish	7.1 ± 6.1	
Child immigrant generation		8.96 ***
Non-Hispanic White ^a^	8.4 ± 6.0	
First generation Hispanics	7.2 ± 6.4	
Second generation Hispanics	7.2 ± 6.1	
Third or higher generation Hispanics	8.0 ± 5.7	
Maximum parent education		6.85 ***
Less than high school	7.0 ± 6.1	
High-school graduate	7.7 ± 6.4	
Some college	8.0 ± 6.2	
College graduate or above	8.3 ± 5.7	
Annual household income		5.57 ***
Less than $20,000	7.3 ± 6.4	
$20,000–35,000	7.3 ± 6.0	
$35,001–50,000	7.3 ± 5.9	
$50,001–75,000	8.3 ± 6.1	
$75,001 or above	8.4 ± 5.7	
Neighborhood socioeconomic status ^b^		5.97 **
Low SES (<−0.40)	7.6 ± 6.3	
Moderate SES (−0.40 to −0.02)	7.3 ± 6.0	
High SES (>−0.02)	8.3 ± 5.8	

^a^ English-speaking, US-born non-Hispanic White children. ^b^ Neighborhood socioeconomic status was based on American Community Survey 5 year estimates of 2009–2013. We categorized this variable into three groups by tertiles. Higher tertiles represent higher socioeconomic status. ^c^ SD indicates standard deviation. * *p* < 0.05; ** *p* < 0.01; *** *p* < 0.001.

**Table 3:** Results of multilevel modeling assessing associations between child language at home and physical activity (times per week), n = 2749.

Predictors	Model 1			Model 2			Model 3		
	B	SE	95% CI	B	SE	95% CI	B	SE	95% CI
Child age									
4–6 years (reference)									
7–9 years	0.36	0.32	−0.26, 0.99	0.37	0.32	−0.25, 1.00	0.41	0.32	−0.22, 1.03
10–12 years	0.78 *	0.33	0.13, 1.42	0.74 *	0.33	0.09, 1.39	0.78 *	0.33	0.13, 1.43
13–15 years	−0.45	0.41	−1.25, 0.34	−0.51	0.41	−1.31, 0.29	−0.51	0.41	−1.30, 0.29
Child gender									
Boy (reference)									
Girl	−0.65 **	0.22	−1.09, −0.21	−0.65 **	0.22	−1.09, −0.21	−0.65 **	0.22	−1.09, −0.21
Child language at home									
Non-Hispanic White ^a^ (reference)									
Hispanics speaking English	−0.74	0.52	−1.76, 0.27	−0.62	0.53	−1.65, 0.41	0.62	1.89	−3.64, 3.76
Hispanics speaking English and Spanish	−1.38 ***	0.31	−1.98, −0.77	−0.94 *	0.37	−1.68, −0.21	−4.78 ***	1.05	−6.84, −2.72
Hispanics speaking Spanish	−1.66 ***	0.39	−2.43, −0.89	−1.24 **	0.45	−2.11, −0.36	−3.49 *	1.39	−6.22, −0.77
Maximum parent education									
Less than high school				−0.28	0.43	−1.12, 0.57	−0.35	0.43	−1.19, 0.49
High-school graduate				0.36	0.43	−0.48, 1.20	0.24	0.43	−0.60, 1.08
Some college				0.18	0.36	−0.54, 0.89	0.09	0.36	−0.62, 0.80
College graduate or above (reference)									
Annual household income									
Less than $20,000 (reference)									
$20,000–35,000				0.03	0.33	−0.63, 0.68	0.01	0.33	−0.64, 0.67
$35,001–50,000				−0.22	0.41	−1.01, 0.58	−0.16	0.40	−0.96, 0.63
$50,001–75,000				0.43	0.45	−0.46, 1.32	0.44	0.45	−0.44, 1.33
$75,001 or above				0.36	0.44	−0.50, 1.22	0.49	0.44	−0.37, 1.35
Child-perceived social environment ^b^				0.35	0.19	−0.01, 0.72	−0.49	0.31	−1.10, 0.12
Neighborhood socioeconomic status ^c^									
Low SES (reference)									
Moderate SES				−0.15	0.36	−0.85, 0.56	−0.12	0.36	−0.82, 0.59
High SES				0.11	0.41	−0.68, 0.91	0.26	0.41	−0.54, 1.06
Child language at home × Social environment									
Non-Hispanic White ^a^ × Social environment (reference)									
Hispanics speaking English × Social environment							−0.30	0.72	−1.71, 1.11
Hispanics speaking English and Spanish × Social environment							1.60 ***	0.40	0.81, 2.39
Hispanics speaking Spanish × Social environment							0.92	0.54	−0.15, 1.98

* *p* < 0.05, ** *p* < 0.01, *** *p* < 0.001. ^a^ The reference group is English-speaking, US-born non-Hispanic White children. ^b^ Social environment was measured based on a four-item scale on neighborhood safety, traffic, and crime. ^c^ Neighborhood socioeconomic status was based on American Community Survey 5 year estimates of 2009–2013. 2013. We categorized this variable into three groups by tertiles. Higher tertiles represent higher socioeconomic status.

**Table 4:** Results of multilevel modeling assessing associations between child immigrant generation and physical activity (times per week), n = 2749.

Predictors	Model 1			Model 2			Model 3		
	B	SE	95% CI	B	SE	95% CI	B	SE	95% CI
Child age									
4–6 years (reference)									
7–9 years	0.37	0.32	−0.26, 0.99	0.38	0.32	−0.24, 1.01	0.41	0.32	−0.21, 1.03
10–12 years	0.79 *	0.33	0.14, 1.43	0.75 *	0.33	0.10, 1.40	0.79 *	0.33	0.15, 1.44
13–15 years	−0.42	0.41	−1.22, 0.37	−0.47	0.41	−1.27, 0.33	−0.46	0.41	−1.26, 0.34
Child gender									
Boy (reference)									
Girl	−0.66 **	0.22	−1.10, −0.22	−0.65 **	0.22	−1.09, −0.21	−0.64 **	0.22	−1.08, −0.20
Child immigrant generation									
Non-Hispanic White ^a^ (reference)									
First generation Hispanics	−1.53 **	0.51	−2.52, −0.53	−1.12 *	0.54	−2.19, −0.05	−6.12 **	1.86	−9.77, −2.46
Second generation Hispanics	−1.46 ***	0.30	−2.04, −0.87	−1.03 **	0.36	−1.75, −0.32	−4.16 ***	1.02	−6.17, −2.16
Third or higher generation Hispanics	−0.59	0.52	−1.62, 0.43	−0.48	0.53	−1.52, 0.56	−0.78	1.84	−4.38, 2.82
Maximum parent education									
Less than high school				−0.27	0.43	−1.11, 0.58	−0.36	0.43	−1.20, 0.49
High-school graduate				0.35	0.43	−0.49, 1.20	0.23	0.43	−0.61, 1.07
Some college				0.16	0.36	−0.55, 0.88	0.07	0.36	−0.64, 0.79
College graduate or above (reference)									
Annual household income									
Less than $20,000 (reference)									
$20,000–35,000				0.01	0.33	−0.65, 0.66	−0.03	0.33	−0.68, 0.63
$35,001–50,000				−0.21	0.41	−1.01, 0.58	−0.20	0.41	−1.00, 0.59
$50,001–75,000				0.41	0.45	−0.48, 1.30	0.41	0.45	−0.48, 1.30
$75,001 or above				0.34	0.44	−0.52, 1.20	0.42	0.44	−0.44, 1.28
Child-perceived social environment ^b^				0.35	0.19	−0.01, 0.71	−0.48	0.31	−1.09, 0.12
Neighborhood socioeconomic status ^c^									
Low SES (reference)									
Moderate SES				−0.15	0.36	−0.86, 0.55	−0.14	0.36	−0.84, 0.57
High SES				0.10	0.41	−0.69, 0.90	0.24	0.41	−0.56, 1.04
Child immigrant generation × Social environment									
Non-Hispanic White ^a^ × Social environment (reference)									
First generation Hispanics × Social environment							2.03 **	0.73	0.60, 3.46
Second generation Hispanics × Social environment							1.29 **	0.39	0.52, 2.05
Third or higher generation Hispanics × Social environment							0.07	0.71	−1.32, 1.47

SES: Socioeconomic status. * *p* < 0.05, ** *p* < 0.01, *** *p* < 0.001. ^a^ The reference group is English-speaking, US-born non-Hispanic White children. ^b^ Social environment was measured based on a four-item scale on neighborhood safety, traffic, and crime. ^c^ Neighborhood socioeconomic status was based on American Community Survey 5 year estimates of 2009–2013. 2013. We categorized this variable into three groups by tertiles. Higher tertiles represent higher socioeconomic status.

**Table 5:** Results of multilevel modeling assessing associations between cultural factors and physical activity (times per week) after multiple imputation, n = 3463 ^a^.

Predictors	Model: Language at Home			Model: Immigrant Generation		
	B	SE	95% CI	B	SE	95% CI
Child-perceived social environment ^b^	−0.12	0.30	−0.71, 0.47	−0.10	0.26	−0.61, 0.41
Child language at home						
Non-Hispanic White ^c^ (reference)						
Hispanics speaking English	0.76	1.47	−2.11, 3.64			
Hispanics speaking English and Spanish	−3.73 ***	1.01	−5.71, −1.75			
Hispanics speaking Spanish	−3.52 **	1.32	−6.10, −0.93			
Child language at home × Social environment						
Non-Hispanic White ^c^ × Social environment (reference)						
Hispanics speaking English × Social environment	−0.50	0.57	−1.62, 0.62			
Hispanics speaking English and Spanish × Social environment	1.16 **	0.39	0.41, 1.92			
Hispanics speaking Spanish × Social environment	0.90	0.51	−0.11, 1.90			
Child immigrant generation						
Non-Hispanic White ^c^ (reference)						
First generation Hispanics				−4.45 *	1.75	−7.89, −1.02
Second generation Hispanics				−3.56 ***	0.86	−5.25, −1.87
Third or higher generation Hispanics				−0.58	1.77	−4.05, 2.89
Child immigrant generation × Social environment						
Non-Hispanic White ^c^ × Social environment (reference)						
First generation Hispanics × Social environment				1.47 *	0.69	0.11, 2.83
Second generation Hispanics × Social environment				1.06 **	0.34	0.40, 1.73
Third or higher generation Hispanics × Social environment				−0.10	0.70	−1.46, 1.27

* *p* < 0.05, ** *p* < 0.01, *** *p* < 0.001. ^a^ All analyses were conducted with adjustment for covariates listed in Table 3 and Table 4. ^b^ The social environment was measured based on a four-item scale on neighborhood safety, traffic, and crime. ^c^ The reference group is English-speaking, US-born non-Hispanic White children.

**Table 6:** Total, direct, and indirect effects of the social environment and physical activity-related social norms on physical activity after bootstrapping.

Pathways	Direct Effect		Indirect Effect		Total Effect	
	B	Bootstrap SE	B	Bootstrap SE	B	Bootstrap SE
Hispanics speaking English and Spanish (n = 987)						
Social environment → physical activity-related social norms	0.30 ***	0.04	-	-	0.30 ***	0.04
PA-related social norms → physical activity	1.09 ***	0.22	-	-	1.09 ***	0.22
Social environment → physical activity	0.79 **	0.28	0.33 ***	0.08	1.12 ***	0.26
First generation Hispanics (n = 180)						
Social environment → physical activity-related social norms	0.33 **	0.11	-	-	0.33 **	0.11
PA-related social norms → physical activity	0.29	0.58	-	-	0.29	0.58
Social environment → physical activity	1.47 *	0.70	0.09	0.19	1.56 *	0.68
Second generation Hispanics (n = 1200)						
Social environment → physical activity-related social norms	0.34 ***	0.04	-	-	0.34 ***	0.04
PA-related social norms → physical activity	1.17 ***	0.21	-	-	1.17 ***	0.21
Social environment → physical activity	0.35	0.26	0.40 ***	0.08	0.74 **	0.25

* *p* < 0.05, ** *p* < 0.01, *** *p* < 0.001. Note: All analyses were conducted with adjustment for covariates listed in Table 3 and Table 4.
